# Nontrivial Replication of Loci Detected by Multi-Trait Methods

**DOI:** 10.3389/fgene.2021.627989

**Published:** 2021-02-03

**Authors:** Zheng Ning, Yakov A. Tsepilov, Sodbo Zh. Sharapov, Zhipeng Wang, Alexander K. Grishenko, Xiao Feng, Masoud Shirali, Peter K. Joshi, James F. Wilson, Yudi Pawitan, Chris S. Haley, Yurii S. Aulchenko, Xia Shen

**Affiliations:** ^1^Biostatistics Group, School of Life Sciences and School of Ecology, Sun Yat-sen University, Guangzhou, China; ^2^Department of Medical Epidemiology and Biostatistics, Karolinska Institutet, Stockholm, Sweden; ^3^Division of Biology, Novosibirsk State University, Novosibirsk, Russia; ^4^Institute of Cytology and Genetics SB RAS, Novosibirsk, Russia; ^5^College of Animal Science and Technology, Northeast Agricultural University, Harbin, China; ^6^Bioinformatics Center, Northeast Agricultural University, Harbin, China; ^7^MRC Human Genetics Unit, MRC Institute of Genetics and Molecular Medicine, University of Edinburgh, Western General Hospital, Edinburgh, United Kingdom; ^8^Centre for Global Health Research, Usher Institute, University of Edinburgh, Edinburgh, United Kingdom; ^9^Kurchatov Genomics Center, Institute of Cytology and Genetics SB RAS, Novosibirsk, Russia; ^10^PolyOmica, 's-Hertogenbosch, Netherlands

**Keywords:** pleiotropy, multivariate analysis, genome-wide association study, cross-phenotype association, replication, genotype-phenotype map

## Abstract

The ever-growing genome-wide association studies (GWAS) have revealed widespread pleiotropy. To exploit this, various methods that jointly consider associations of a genetic variant with multiple traits have been developed. Most efforts have been made concerning improving GWAS discovery power. However, how to replicate these discovered pleiotropic loci has yet to be discussed thoroughly. Unlike a single-trait scenario, multi-trait replication is not trivial considering the underlying genotype-multi-phenotype map of the associations. Here, we evaluate four methods for replicating multi-trait associations, corresponding to four levels of replication strength. Weak replication cannot justify pleiotropic genetic effects, whereas strong replication using our developed correlation methods can inform consistent pleiotropic genetic effects across the discovery and replication samples. We provide a protocol for replicating multi-trait genetic associations in practice. The described methods are implemented in the free and open-source R package MultiABEL.

## Introduction

During the past decade, single-trait genome-wide association studies (GWAS) have successfully identified a multitude of genetic variants underlying complex traits (Visscher et al., 2017). However, the effects of the genetic variants, such as single-nucleotide polymorphisms (SNPs), on complex traits are usually very small. This directly limits the discovery power in most GWASs. Given wide-spread pleiotropy (Watanabe et al., 2019) and aiming to improve power, many multi-trait analysis methods have been developed in recent years to jointly analyze multiple correlated phenotypes. At the early stage, most multi-trait tools were based on individual-level data. For example, the –multivariate module of PLINK implements canonical correlation analysis (CCA) to identify the association between each SNP and linear combinations of phenotypes (Ferreira and Purcell, 2008); Combined-PC (Aschard et al., 2014) performed a principal components analysis on the phenotype data to improve statistical power. By combining a linear mixed model and multivariate analysis of variance (MANOVA), a multi-trait analysis was also shown to be effective for omics measurements (Shen et al., 2017).

As many GWAS have been performed in different study cohorts or populations, given the difficulty of sharing and combining individual-level data, multi-trait methods based on GWAS summary-level data became popular. Many such methods have been developed and demonstrated their benefits in boosting discovery power (Cotsapas et al., 2011; Solovieff et al., 2013; van der Sluis et al., 2013; Kim et al., 2015). For example, Stephens (2013) outlined a unified multivariate analysis framework based on Bayesian model comparisons; Zhu et al. (2015) introduced two test statistics *S*_*Hom*_ and *S*_*Het*_ to improve statistical power under different assumptions of effect sizes in which seven additional loci were suggested by jointly analyzing the summary statistics of three traits from the GIANT consortium (Park et al., 2016). A simulation study (Porter and O'Reilly, 2017) demonstrated that the statistical powers of most methods are similar to the power of the standard MANOVA. There are multi-trait methods where, although the information of multiple traits is used, the discoveries are yield from a univariate test. For example, GenomicSEM investigates the association between an SNP and a latent factor defined by several phenotypes (Grotzinger et al., 2019). MTAG (Multi-trait analysis of GWAS) was developed to integrate GWAS summary results of multiple related traits and improve the inference in each single-trait GWAS (Turley et al., 2018). In this perspective paper, we limit the term “multi-trait methods” to the methods where a multivariate test statistic is applied.

For any scientific discovery, replication—the ability to reproduce the findings of an original study in an independent experiment—plays a key role in the evaluation of credibility (Randall and Welser, 2018). Although many multi-trait methods were proposed and were demonstrated to boost genetic association discovery power, the multi-trait replication strategy has yet to be agreed upon. When an SNP is discovered in multi-trait analysis, a commonly used approach for replication is to replicate the associations trait-by-trait in a replication sample (Liang et al., 2017; Gialluisi et al., 2019). However, there are at least two disadvantages of such “univariate” replication: firstly, when the number of tested traits is large, multiple testing arises when determining the replication significance threshold; secondly, univariate replication does not account for phenotypic correlations between the tested traits, which generates conservative significance threshold after correction for multiple testing. Another straightforward way for replication is to directly perform the multi-trait test in a replication sample and see whether the overall association (omnibus *p*-value) is significant (Karnes et al., 2017; Liang et al., 2017; Luo et al., 2020). Although this strategy provides a unified test statistic, it does not reveal whether the effects that locus exhibits on traits in the discovery are the same (or similar) as those observed in replication. Thus, the consistency of the multiple genetic effects between the discovery and replication samples is usually overlooked. Even if the genetic effect sizes and directions are distinct between the discovery and replication samples, the multivariate replication test may still show significance.

Aiming to develop a stronger replication criterion, Shen et al. (2017) suggested a phenotype score replication strategy. For a discovered SNP, the strategy in the discovery sample is as follows: construct a phenotype score that best fits the SNP genotype data; then, in the replication sample, compute the corresponding phenotype score and test the association between the phenotype score and the SNP genotype in the replication sample. In this way, the phenotype score replication provides a signed test statistic, which conceptually replicates the overall direction of the cross-phenotype effects. However, this replication strategy has not been implemented using GWAS summary statistics, and its performance has not been systematically examined.

To verify a locus detected by multi-trait methods, the first task is to replicate the association between the locus and the set of traits tested in the discovery phase. The next task is to evaluate the consistency of multi-trait genetic associations between discovery and replication samples across the traits in question, which calls for stronger replication strategies. To accomplish these two tasks in the replication of multi-trait signals, in this perspective article, we investigated and compared the performance of four replication methods. We began by reviewing the MANOVA and phenotype score, which we suggest as methods for locus replication. Then, we evaluated the consistency of effects that the locus exhibits onto multiple traits in question, i.e., replication of multivariate effects. A discovery study may have enough power to establish the overall association between a locus and a set of traits but insufficient power to robustly estimate all the effects the locus exhibits on multiple traits. In this case, one would expect that a locus would pass a locus-level replication but would fail to pass the multivariate genetic effects replication. To evaluate the similarity between estimates of multivariate genetic effects in discovery and replication, we introduced a Monte-Carlo (MC)-based correlation method. Through simulations of different scenarios, we have illustrated the strength and complementarity of four methods that cover different aspects of multivariate replication: (i) MANOVA, as a representative of multivariate methods providing unsigned omnibus *p*-values; (ii) phenotype score replication; (iii) Pearson correlation method; and (iv) Kendall correlation method. The implementation of these methods only requires summary association statistics. We suggest a four-level replication strategy where the above four replication methods are applied sequentially. To demonstrate the application of the four-level replication strategy, we studied the SNPs discovered by MANOVA, using the GWAS summary statistics from the GIANT consortium as an example, and try to replicate them in the UK Biobank (UKB).

## Summary of the Methods

### MANOVA

It has been shown that MANOVA can be performed using summary statistics (Stephens, 2013; Zhu et al., 2015). To simplify the formulae, we assume the phenotypes are standardized to have mean zero and variance one, and genotypes are centered to have mean zero. *k* traits *Y*_1_, ..., *Y*_*k*_ are dependent variables. If we denote the true marginal effects of a SNP on the *k* traits by ***β***, then the null hypothesis in MANOVA is *H*_0_:***β*** = **0**. Let $\text{t}={\left[t_{1},...,t_{k}\right]}^{\prime }$ be the vector of single-trait t-test statistics from association tests between the genotypes **g** of a single SNP and the *k* phenotypes, and R^*^ ≡ Cor(**t** ∣ ***β***) = Var(**t** ∣ ***β***). If R^*^ is available, the test statistic

(1) $$\begin{array}{l} T^{2}={\text{t}}^{\prime }{\text{R}}^{*-1}\text{t}, \end{array}$$

asymptotically follows a χ^2^ distribution with *k* degrees of freedom under the null hypothesis. In practice, an unbiased estimate of R^*^ can be obtained by selecting a large number of independent variants from the meta-GWAS summary statistics and calculating their correlation coefficients (Stephens, 2013; Zhu et al., 2015). More details can be found in Material and Methods and Supplementary Note.

### Phenotype Score

If individual-level data are available, given an SNP, we can use CCA to get its most associated linear combination of traits. This linear combination of traits, which we name as phenotype-score, can be treated as a new phenotype. It has been shown (O'Reilly et al., 2012) that the coefficients in CCA are equivalent to the estimate of **b**_*k*×1_ in this reversed multiple regression

$$\begin{array}{l} \text{g}=\text{Y}\text{b}+\epsilon , \end{array}$$

where **g**_*n*×1_ and **Y**_*n*×*k*_ represent genotypes and phenotypes respectively. Assuming Hardy-Weinberg equilibrium (HWE), $\hat{\text{b}}$, which is the estimate of **b**, can be obtained by

$$\begin{array}{l} \hat{\text{b}}=2f\left(1-f\right)\cdot {\hat{\text{R}}}^{*-1}\hat{\beta }, \end{array}$$

where *f* is the coding allele frequency of the SNP (Supplementary Note). Therefore, we can get $\hat{\text{b}}$ based on summary statistics and construct the phenotype-score $\text{S}=\text{Y}\hat{\text{b}}$. Taking **S** as a new phenotype and denoting the effect of the SNP on **S** as β_*s*_, we can estimate and test β_*s*_ in the discovery and replication populations (Supplementary Note). If ${\hat{\beta }}_{s}$ is significantly different from 0 and has the same sign in both populations, then we consider the association between the SNP and the phenotype-score is replicated.

### Correlation Methods

Aiming to evaluate the similarity between estimates of multivariate genetic effects from discovery and those from replication samples across multiple traits, we developed this MC-based correlation method. The key idea is to evaluate the similarity of marginal effects with the uncertainty in estimates taken into account. Let $\hat{\beta }=\left({\hat{\beta }}_{1},{\hat{\beta }}_{2},...,{\hat{\beta }}_{k}\right)$ be the vector of estimated marginal effects across the *k* phenotypes. Then $\text{Cor}\left(\hat{\beta }|\beta \right)\approx {\text{R}}^{*}$ (Supplementary Note). Because the variances of $\left\{{\hat{\beta }}_{i}\right\}$ are available, we can obtain $\hat{\Sigma }$, which is an estimate of $\Sigma =\text{Cov}\left(\hat{\beta }|\beta \right)$. This allows us to draw $\beta_{disc}^{MC}$ from $N\left(\hat{\beta },{\hat{\Sigma }}_{disc}\right)$ for the discovery sample, and $\beta_{rep}^{MC}$ from $N\left(\hat{\beta },{\hat{\Sigma }}_{rep}\right)$ for the replication sample. Then we can compute their correlation coefficients. Here, we compute (i) Pearson's correlation coefficient:

$$\begin{array}{l} \rho_{\beta }^{MC}=\frac{\overset{k}{\underset{j=1}{\sum }}\left(\beta_{j,disc}^{MC}-{\bar{\beta }}_{disc}^{MC}\right)\left(\beta_{j,rep}^{MC}-{\bar{\beta }}_{rep}^{MC}\right)}{\sqrt{\overset{k}{\underset{j=1}{\sum }}{\left(\beta_{j,disc}^{MC}-{\bar{\beta }}_{disc}^{MC}\right)}^{2}}\sqrt{\overset{k}{\underset{j=1}{\sum }}{\left(\beta_{j,rep}^{MC}-{\bar{\beta }}_{rep}^{MC}\right)}^{2}}} \end{array}$$

and (ii) Kendall's rank correlation coefficient

$$\begin{array}{l} \tau_{\beta }^{MC}=\frac{2}{k\left(k-1\right)}\underset{j<j^{\prime }}{\sum }\text{sgn}\left(\beta_{j,disc}^{MC}-\beta_{j^{\prime },disc}^{MC}\right)\cdot \text{sgn}\left(\beta_{j,rep}^{MC}-\beta_{j^{\prime },rep}^{MC}\right), \end{array}$$

which measures the ordinal association between $\beta_{disc}^{MC}$ and $\beta_{rep}^{MC}$. Both correlation coefficients measure the similarity of estimates of genetic effects between discovery and replication samples across multiple traits. However, compared to Kendall's correlation, Pearson's correlation gives more weight to those associations with large effect sizes. In other words, if an SNP only affects few traits but those with large effect sizes, Kendall's correlation is likely to be close to 0, but Pearson's correlation may not be. By performing parametric bootstrap simulations, we can obtain estimated distributions of ρ_β_ and τ_β_. Based on this distribution, the parametric bootstrap confidence intervals (CI) are attained, which can be used to evaluate the similarity between estimates of multivariate genetic effects.

## Simulations

We performed a series of simulations to compare the replication results of four methods: MANOVA, phenotype score, Pearson correlation, and Kendall correlation. Four different scenarios of cross-phenotype effects were simulated: (i) null, where the SNP did not affect any trait; (ii) unmatched multi-trait effects, where the SNP exhibited effects on all traits, but the effects were independently simulated for discovery and replication samples; (iii) matched single-trait effect, where the SNP affected only one trait, and the true effect was the same in the discovery and replication samples; and (iv) matched multi-trait effects, where the SNP had effects on all the traits, and the true effects were the same in the discovery and replication samples. Two different numbers of traits, 6 or 32, were simulated. In practice, the phenotypic correlation matrices between the discovery and replication samples can be consistent or inconsistent, e.g., when estimated from two samples with different sample overlaps among the phenotypes. Its potential impact on replication was also evaluated. In the simulations, the phenotypic correlation matrices were based on the true observed correlation matrices in the GIANT and UKB data. More details can be found in Supplementary Note.

**Figure 1:**
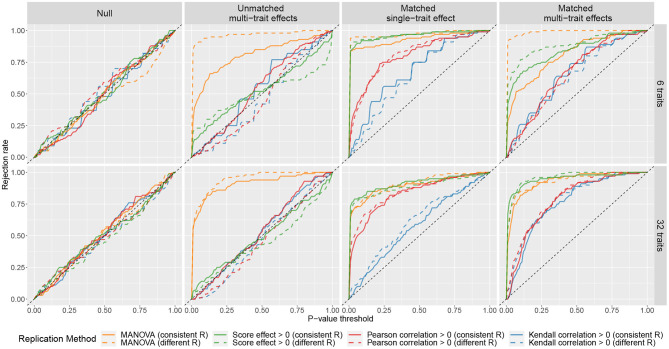
Rejection rates of four replication methods at different *P*-value thresholds under different simulated scenarios. For each scenario, 100 simulations were performed. In each simulation, a discovery sample and a replication sample were generated with one SNP and either 6 traits (the first row) or 32 traits (the second row). The *k* traits were simulated as **y** = ***β****g* + ***ϵ***, where ***β*** = (β_1_, ..., β_*k*_) represents the cross-phenotype effects of the SNP on the *k* traits, and ***ϵ*** = (ϵ_1_, ..., ϵ_*k*_) are residuals. Four matching scenarios between the cross-phenotype effects in discovery sample ***β***_*disc*_ and those in replication sample ***β***_*rep*_ were simulated: (i) null, where ***β***_*disc*_ = ***β***_*rep*_ = **0**; (ii) unmatched multi-trait effects, where ***β***_*disc*_ and ***β***_*rep*_ were independently drawn from a normal distribution; (iii) matched single-trait effect, where β_1,*disc*_ was drawn from a normal distribution and β_1,*rep*_ = β_1,*disc*_, while (β_2,*disc*_, ..., β_*k,disc*_) = (β_2,*rep*_, ..., β_*k,rep*_) = **0**; and (iv) matched multi-trait effects, where ***β***_*disc*_ was drawn from a normal distribution and ***β***_*rep*_ = ***β***_*disc*_. The covariance matrices of ***ϵ*** in discovery sample and replication sample can be either the same (marked as consistent R) or different (marked as different R). More details can be found in Supplementary Note.

The results of the simulations are summarized in Figure 1, where the curves showed the rejection rates at different *P*-value thresholds. The curves of MANOVA were above the diagonal in scenarios (ii), (iii), and (iv). This means with MANOVA, an SNP is considered replicated as long as it affects at least one trait in the replication sample no matter how the cross-phenotype effect compares to that in the discovery sample. The curves of phenotype score replication did not deviate much from the diagonal in scenarios (ii). It shows when there is a large discrepancy in the cross-phenotype effect between the samples; the phenotype score does not consider it as replicated. However, the curves of phenotype score replication were above the diagonal in scenario (iii). So even when the SNP affects only one trait, the association between the SNP and the phenotype score can still be replicated. When there were 32 traits, the curves of the Kendall correlation obviously deviated from the diagonal in scenario (iv) but only slightly deviated from the diagonal in scenario (iii). This indicates that Kendall correlation replication is more specific for confirming the cross-phenotype effect where an SNP affects multiple traits. The performance of the Pearson correlation is similar to that of the Kendall correlation; the exception is in scenario (iii), where the curves of the Pearson correlation were between those of the Kendall correlation and those of the other two methods. Although the correlation methods were more specific to evaluate the pattern of the multi-trait effects, their curves were under those of MANOVA and phenotype score, which indicates lower sensitivity.

## Four-Level Replication Strategy for Associations Detected by Multi-Trait Methods

**Figure 2:**
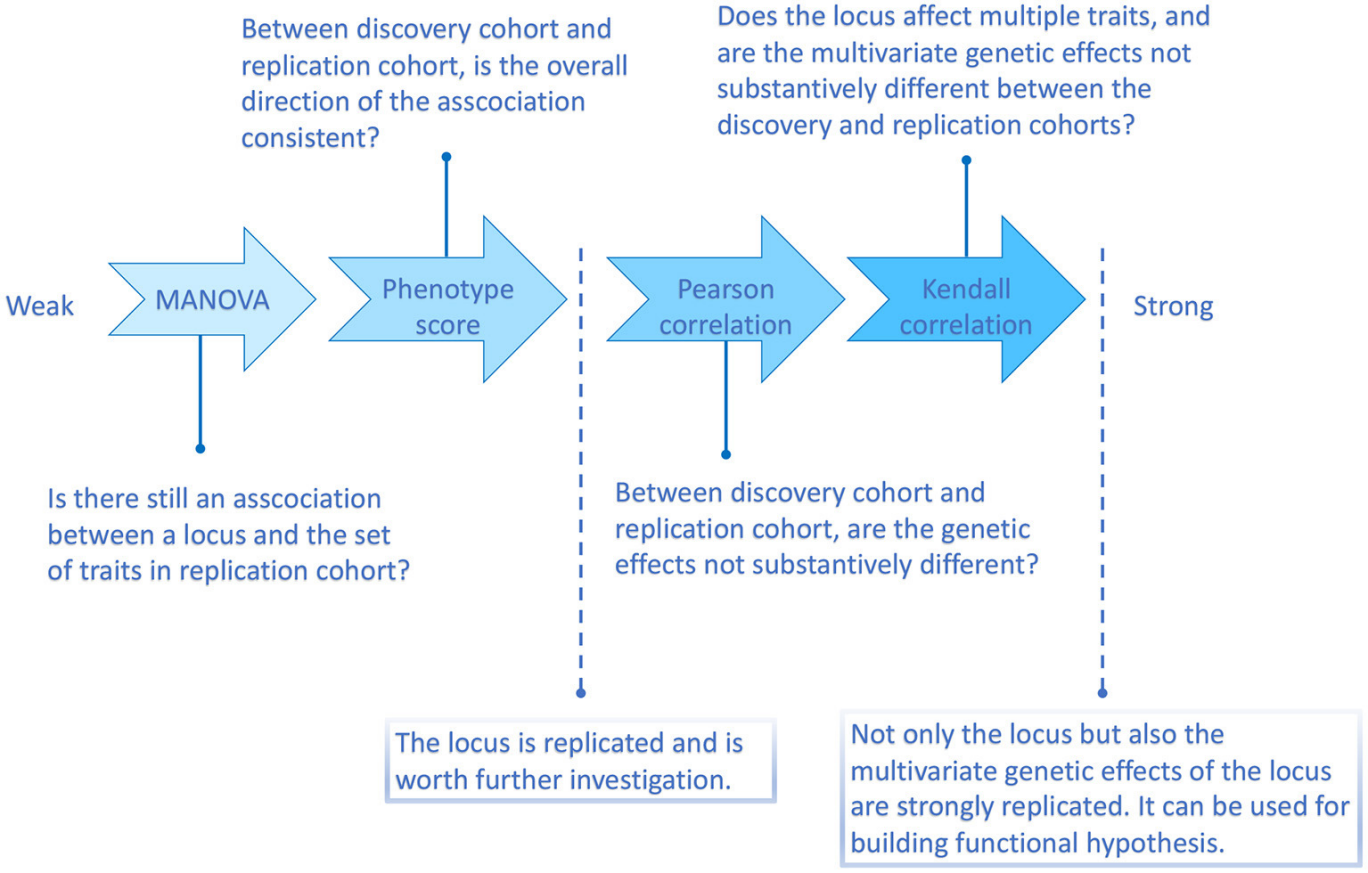
Flowchart illustrating the four multi-trait replication methods and the questions they can answer.

According to the above simulation results, the four methods are with different replication strength. Therefore, we suggest the following four-level replication strategy, summarized in Figure 2.

###  Level 1: Omnibus *p*-Value Test for Locus Replication

Omnibus *p*-value test (including MANOVA and many other multivariate methods) verifies whether there is indeed an association between the discovered locus and the set of traits in a replication sample. As long as the association is present in the replication sample, the locus is considered as replicated regardless of whether the multivariate genetic effects are similar between discovery and replication or not. This is the weakest type of multivariate replication.

###  Level 2: Phenotype Score for Locus Replication

The phenotype score method (Shen et al., 2017) can filter out some inconsistent cross-phenotype associations, such as scenario (ii) in the simulation, where the directions of the associations were distinct in discovery and replication samples. The score replication is stronger than MANOVA replication and is similar to a classical univariate replication in that it asks for both experiment-wise significance and consistency of effects between discovery and replication. However, in a univariate case, consistency is defined as and justified by the same sign of the effect of an associated allele. In contrast, the phenotype score operates in many dimensions and can be dominated by a small number of traits, which precludes it from evaluating the overall similarity of estimates of genetic effects a locus exhibits on the tested traits. Thus, if a locus has passed the score replication, we can consider it an established locus. However, we can not be sure about the quality and stability of the locus-phenotype map suggested by the discovery study. The next steps aim to judge how well we can replicate the vector of effects.

###  Level 3: Pearson Correlation Method for Replication of Multivariate Genetic Effects

Pearson correlation method is a bridge between the phenotype score and the Kendall correlation method. As a correlation test strategy, each tested trait contributes more than it does in the phenotype score method. However, Pearson's correlation is more responsive to large genetic effects than Kendall's correlation. Therefore, when an SNP only affects few traits with similarly large effect sizes in both discovery and replication samples, such as scenario (iii) in the simulation, the Pearson correlation method would consider the multivariate genetic effects similar.

###  Level 4: Kendall Correlation Method for Replication of Multivariate Genetic Effects

Kendall correlation method is the strongest one among the four methods. Since Kendall's correlation is based on the ranks of estimated marginal genetic effects, any factor disturbing the ranks would weaken the correlation. For example, if an SNP does not affect all the tested traits, then its estimated marginal effects around zero on those irrelevant traits will be randomly ranked, which reduces Kendall's correlation (Supplementary Figure 1). An extreme case is when an SNP associates with only one trait. In this case, there will be almost no similarity of the estimated effect sizes rank between samples. Therefore, if an SNP can be replicated by the Kendall correlation method, it is more likely to be associated with multiple traits, and the effect sizes (ranks) are consistent.

## Example: UKB Replication of Anthropometric Loci Discovered by MANOVA in the GIANT Consortium

In order to compare the performance of different replication methods using real data, we investigated the loci discovered in the GIANT consortium and replicated them in UKB by MANOVA. We used six anthropometric traits for multi-trait analysis: BMI, height, weight, hip circumference (denoted here as HIP), waist circumference (WC), and waist-to-hip ratio (WHR). Among the 2,348,642 SNPs in the GIANT data, 21,240 SNPs had MANOVA *p*-value <5 × 10^−8^. Considering SNPs <1 Mb distant from most significant SNP as one locus, the 21,240 SNPs were clumped into 449 loci. For each locus, we defined its top SNP as the SNP with the smallest MANOVA *p*-value in GIANT. After, we used the top SNPs to represent loci.

A total of 317 out of the 449 top SNPs could be replicated in UKB with a MANOVA *p*-value threshold of 0.05/449. To illustrate the performance of different replication methods, among the 317 loci, we looked into 24 “multivariate-only” loci as an example. These 24 loci could be discovered and replicated by MANOVA, but none of them could reach *p*-value <5 × 10^−8^ in univariate GWAS for any of the six anthropometric traits. The replication results were summarized in Supplementary Table 1.

According to the results from the phenotype score method, all except two SNPs had a *p*-value < 0.0021 (experiment-wise *P* < 0.05 after Bonferroni correction for 24 tests) in UKB. Additionally, all of their ${\hat{\beta }}_{s}$ had the same sign in both GIANT and UKB. The results suggest that, for any of the 22 SNPs, there is no large discrepancy in its multi-trait association between GIANT and UKB.

Results from correlation methods indicated different levels of similarity of multivariate genetic effects between discovery and replication across the 22 loci. Both the two correlation methods identified the same 19 SNPs for which the pattern of multi-trait association was significantly similar between the discovery and replication samples. For some loci, the lower bounds of their 95% confidence intervals (CI) were higher than the others, suggesting a higher level of similarity of multivariate genetic effects between discovery and replication. Supplementary Figure 2 visualized loci with different levels of similarity. For instance, although both rs2138275 and rs4968164 could be replicated by MANOVA, the 95% CI of τ_β_ was [0.73, 1] for rs2138275, and [−0.07, 1] for rs4968164. This means the multi-trait marginal genetic effects across samples were similar for rs2138275 but not for rs4968164. The contrast indicated that the underlying six-trait cross-phenotype association was more plausible at the locus led by rs2138275. For the locus led by rs4968164, although it could be detected and replicated by MANOVA, the SNP might be associated with only a small subset of the six traits.

Because the association of a locus with multiple traits is necessary but not sufficient for pleiotropy (Solovieff et al., 2013), it is more promising to discover pleiotropy from loci with reliable associations to multiple traits. We hypothesized that the loci with significant results in the Kendall correlation method were more likely to be associated with more traits in general, even those not limited to anthropometric measurements. According to the results based on the records in PhenoScanner (Staley et al., 2016), indeed the loci supported by the Kendall correlation method tended to be associated with more traits than the others (Supplementary Figure 3).

## Discussion

For signals discovered in multivariate analysis, a more systematic replication strategy is necessary to better evaluate the credibility of these signals. In this perspective, we introduced a four-level replication strategy for replication of loci detected by multi-trait methods. The replication strategy only requires summary association statistics, which are straightforward to apply to multi-trait GWAS analyses. Our results show that the four methods are complementary and provide different degrees of replication strength.

In the strategy proposed (Figure 2), to verify a locus detected by multi-trait methods, in the first phase we replicate the association between the locus and the set of traits tested in the discovery phase (MANOVA and phenotype score); in the next phase, we evaluate the consistency of multi-trait genetic associations between discovery and replication across the traits in question (Pearson and Kendall correlations). When only the MANOVA test is passed, this is the weakest type of replication, under which we have formally reached the *p*-value low enough (usually, experiment-wise *p* < 0.05) to claim replication of an association. This criterion is, however, weaker than that conventionally used in single-trait GWAS in which we ask not only for significance but also for consistency. The requirement of overall consistency is reached with the phenotype score replication step. At this stage, we can consider the locus to be established, that is, we can claim consistent and significant association of the locus to (at least some of the) investigated traits. The question of whether the discovery phase was informative enough to establish the genotype-multi-phenotype map of the associations of the locus is addressed in the second phase. In principle, given score replication was passed, one does expect that the Pearson correlation between effects estimated in discovery and replication will be positive. However, it may be weak, meaning that a researcher can not trust individual estimates of effects. In such a situation, any attempt to utilize observed associations of individual traits, e.g., the benefit of functional interpretation, is likely doomed. However, the Pearson correlation may be high because the locus exhibits a strong effect on just a few—or even only one—traits in the set or because it exhibits effects of many traits in the set. The fourth method, the Kendall correlation, allows one to distinguish between the two scenarios (see Figure 2).

To detect which traits are more likely to be irrelevant for an SNP, we can compare the ordinal discordance generated by each trait in Kendall's correlation. For an SNP, if the effects of the locus on a trait introduce much discordance, then the association between the SNP and the trait would be more suspicious. For example, the marginal effect of height generated much discordance for rs11231693, which indicated that height was more likely not contributing to the multivariate association between rs11231693 and the group of traits. This could be verified by the non-significant association in height GWAS (*p*-value was 0.91 in GIANT2015 and 0.96 in UKB).

In the simulation, as we aim to replicate multi-trait genetic effects, for a good replication method, the performance should not be driven by whether the phenotypic correlation matrices are the same between the discovery and replication cohort. However, we found that for MANOVA, replication samples with a different phenotypic correlation structure (“different **R**”) could perform better than the “consistent **R**” scenario in the six traits settings. The phenomenon can be attributed to the choice of **R** for the replication cohort. In the six traits setting, **R** was from GIANT for the discovery cohort. Therefore, for “consistent **R**,” **R** for the replication cohort was also from GIANT; whereas for “different **R**,” **R** was from UKB instead. The *T*^2^ statistic for MANOVA based on **R** from UKB could be on average larger than those based on **R** from GIANT, as for MANOVA, the replication results are fully determined by the replication cohort without comparing with the multi-trait genetic effects in the discovery cohort.

With our results, we emphasize the value of various replication strategies for multi-trait analysis. Proper replicated results from the multivariate analysis may substantially enhance our understanding of shared genetic architecture between complex traits and disease and reveal interesting biological knowledge.

The developed methods are implemented and freely available in the R package **MultiABEL**: https://github.com/xiashen/MultiABEL).

## Materials and Methods

###  Estimation of R^*^

Let **R** represent the phenotypic correlation matrix of the *k* phenotypes. According to Zhu et al. (2015), R^*^ ≈ **R** when the phenotypes are measured on the same set of individuals. If the individuals for trait *j* and those for trait *j*′ partially overlap, we denote the number of overlapping individuals as *n*_0_, those with trait *j* but not trait *j*′ as *n*_1_, and those with trait *j*′ but not trait *j* as *n*_2_. We then have

(2) $$\begin{array}{l} R_{j,j^{\prime }}^{*}=\text{Cor}\left(t_{j},t_{j^{\prime }}|\beta \right)\approx \frac{n_{0}}{\sqrt{\left(n_{0}+n_{1}\right)\left(n_{0}+n_{2}\right)}}R_{j,j^{\prime }} \end{array}$$

(Supplementary Note). Therefore, the correlation of t-statistics is a shrinkage version of the phenotypic correlation, with a factor determined by the level of overlap. Because (2) holds for all SNPs, an unbiased estimate of the correlation matrix R^*^ can be obtained by selecting a large number of independent variants from the meta-GWAS summary statistics and calculating their correlation coefficients (Stephens, 2013; Zhu et al., 2015).

For correlation matrix estimation, we used previously proposed approach based on correlation of Z-statistics between independent unassociated SNPs (Zhu et al., 2015). We filtered SNPs based on given criteria: MAF > 0.1; high imputations quality as indicated either by INFO > 0.99 (for UKB) or by *N*_*e*_/*N* > 0.9, where $N_{e}=1/\left(2pq{\left(se\right)}^{2}\right)$, for GIANT; |*Z*| < 2; the sample of 200,000 independent (LD pruned) SNPs to compute correlation matrix (the list of SNPs was obtained by—“prune” option for PLINK using 1,000 Genomes data). In total, 128,670 independent SNPs were used to estimate the correlation matrix for GIANT and 166,000 SNPs for UKB. All estimated correlation matrices can be found in Supplementary Table 2.

###  Discovery Sample: GIANT

We downloaded the summary association statistics of six anthropometric traits meta-GWAS by the GIANT consortium from: https://www.broadinstitute.org/collaboration/giant/index.php/GIANT_consortium_data_files. We used six anthropometric traits: BMI, height, weight, hip circumference (denoted here as HIP), waist circumference (WC), and waist-to-hip ratio (WHR). BMI data are from Locke et al. (2015); height data are from Wood et al. (2014); weight data are from Randall et al. (2013); HIP, WC, and WHR data are from Shungin et al. (2015).

As HapMap II allele frequencies were reported in the meta-GWAS instead of pooled allele frequencies across all the cohorts, we excluded SNPs with sample a size <70,000 and MAF <0.01. SNPs with missing allele frequencies were also excluded. All SNPs were merged with genome positions (GRCh37) and filtered for autosomes only and position missings. In total, we ended with 2,348,642 SNPs.

###  Replication Sample: UK Biobank

UKB participants were recruited from the general UK population across 22 centers between 2006 and 2010. Subjects were aged 40–69 at baseline, underwent extensive phenotyping by questionnaire and clinic measurements, and provided a blood sample. Genotyping has been done with the first wave released in July 2015 and the second wave released in July 2018. Because the objective of this study is to demonstrate the replication methods, we use the first wave data of UKB as an example dataset. Data access to UKB was granted under Project No. 14302 and No. 19655. Phenotypes and genotypes were downloaded directly from UKB. In total 502,664 subjects had phenotypic information available, of whom 152,732 had been genotyped, of these 120,286 were identified as genetically British by UKB, of which 118,182 (55,842 men) had completed phenotyping. These subjects were taken forward for analysis.

Participants provided full informed consent to participate in UKB. This study was covered by the generic ethical approval for UKB studies from the NHS National Research Ethics Service (approval letter dated 17th June 2011, Ref 11/NW/0382). The authors in this study were completed blinded to individual-level data collection and preparation. The phenotypes involved in this study were adjusted for age, sex, and batch before being standardized to have mean zero and variance one.

## Data Availability Statement

The original contributions presented in the study are included in the article/Supplementary Material, further inquiries can be directed to the corresponding author/s.

## Author Contributions

XS and YA initiated the study. XS, YA, YP, and CH coordinated the study. ZN, XS, YP, and YA developed statistical methods. ZN and YT performed the data analysis. ZW contributed to the method development. ZW, SS, AG, XF, MS, PJ, and JW contributed to the data analysis. ZN and XS drafted the manuscript. YT, PJ, JW, YP, CH, and YA contributed to the manuscript writing. All authors contributed to the article and approved the submitted version.

## Conflict of Interest

YA was a co-founder and co-owner of private research organizations PolyOmica and PolyKnomics. The remaining authors declare that the research was conducted in the absence of any commercial or financial relationships that could be construed as a potential conflict of interest.
