# Supplementary material for: Nontrivial Replication of Loci Detected by Multi-Trait Methods
Source: Front Genet. 2021 Feb 3;12:627989. doi: 10.3389/fgene.2021.627989 (PMC7886991; doi:10.3389/fgene.2021.627989)
Supplement: Supplementary file 1 [file Presentation_1.pdf]

Supplementary Material

1 SUPPLEMENTARY NOTE

1.1 Complete methods and derivations

Multi-trait association modeling and hypothesis testing

To simplify the formulae, we assume the phenotypes are standardized to have mean zero and variance one, and genotypes are centered to have mean zero. k traits $Y_1, ...Y_k$ are dependent variables in MANOVA. Now we have n individuals with these k phenotypes $\mathbf{Y}_{n\times k}$ and a biallelic marker $\mathbf{g}_{n\times 1}$. Then the association between the group of k phenotypes and the marker can be expressed as a multivariate regression

$$\mathbf{Y}_{n\times k} = \mathbf{g}_{n\times 1}\boldsymbol{\beta}'_{k\times 1} + \mathbf{e}_{n\times k}, \tag{S1}$$

which can be tested via MANOVA for the null hypothesis

$$H_0 : \boldsymbol{\beta} = \mathbf{0}.$$

The estimates in the vector $\hat{\boldsymbol{\beta}}$ are known from GWA summary statistics. Below, we show how a MANOVA test statistic can be obtained without knowing the original data.

Calculating the multi-trait association test statistic

First of all, it is known that MANOVA test statistics, such as Pillai's trace, Wilk's lambda, etc., are all equivalent to an F statistic for a single factor analysis (Olson, 1976), which is what we conduct in GWAS. When sample size is large, the F test can be approximated by a χ^2 test. Let $\mathbf{t} = [t_1, ..., t_k]'$ be the vector of single-trait t-test statistics across the k phenotypes on the marker $\mathbf{g}$, and $\mathbf{R}^* \equiv \text{Cor}(\mathbf{t}) = \text{Var}(\mathbf{t})$. If $\mathbf{R}^*$ is available, the test statistic

$$T^2 = \mathbf{t}'\mathbf{R}^{*-1}\mathbf{t}, \tag{S2}$$

which asymptotically follows a χ^2 distribution with k degrees of freedom under the null hypothesis.

Let $\mathbf{R}$ represent the phenotypic correlation matrix of the k phenotypes. According to Zhu et al. (2015), $\mathbf{R}^* = \mathbf{R}$ when the phenotypes are measured on the same set of individuals. When the samples partially overlap across different phenotypes, we derive the theory of shrinkage phenotypic correlation matrix below, which links $\mathbf{R}$ and $\mathbf{R}^*$. With the relationship between $\mathbf{R}$ and $\mathbf{R}^*$, $\mathbf{R}^*$ can be estimated using summary association statistics.

Shrinkage estimate of the phenotypic correlation matrix

Given a specific variant in GWAS, for trait j and trait j', let $\mathbf{g}$ be the genotypes of the overlapping n_0 individuals between the two traits. For the n_1 individuals with only trait j, we denote their genotypes as $\mathbf{x}$; and for the n_2 individuals with only trait j', we denote their genotypes as $\mathbf{z}$. In another word, the genotypes are $[\mathbf{g}', \mathbf{x}']'$ for trait j, and $[\mathbf{g}', \mathbf{z}']'$ for trait j'. If the cohorts for trait j and trait j' are random samples of the same population, we can assume the variances of the variant are same and denote it as σ_g^2. Assuming

$\bar{\mathrm{g}} = \bar{\mathrm{x}} = \bar{\mathrm{z}} = 0$, we have

$$\frac{\mathbf{g}'\mathbf{g}}{n_0} \approx \frac{\mathbf{x}'\mathbf{x}}{n_1} \approx \frac{\mathbf{z}'\mathbf{z}}{n_2} \approx \sigma_g^2 \tag{S3}$$

Let $t_j = \hat{\beta}_j / \sqrt{\hat{\sigma}^2_{\hat{\beta}_j}}$ and $t_{j'} = \hat{\beta}_{j'} / \sqrt{\hat{\sigma}^2_{\hat{\beta}_{j'}}}$ be the test statistics of phenotypes j and j' against the variant, σ_j^2 and $\sigma_{j'}^2$ be the phenotypic variances, we have

$$t_j = \frac{\hat{\beta}_j}{\sqrt{\hat{\sigma}^2_{\hat{\beta}_j}}} = \frac{(\mathbf{g}'\mathbf{g} + \mathbf{x}'\mathbf{x})^{-1} [\mathbf{g}', \mathbf{x}'] \boldsymbol{y}_j}{\sqrt{\hat{\sigma}^2_{r_j} (\mathbf{g}'\mathbf{g} + \mathbf{x}'\mathbf{x})^{-1}}} \approx \frac{[\mathbf{g}', \mathbf{x}'] \boldsymbol{y}_j}{\sqrt{\sigma_j^2 \sigma_g^2 (n_0 + n_1)}},$$

where $\hat{\sigma}^2_{r_j}$ is the estimated residual variance in univariate regression. As the effect of a single variant is usually small, we can approximate $\hat{\sigma}^2_{r_j}$ by σ_j^2. Similarly,

$$t_{j'} \approx \frac{[\mathbf{g}', \mathbf{z}'] \boldsymbol{y}_{j'}}{\sqrt{\sigma_{j'}^2 \sigma_g^2 (n_0 + n_2)}}$$

Therefore,

$$\begin{aligned} R^*_{j,j'} &= \mathrm{Cor}(t_j, t_{j'}) = \mathrm{Cov}(t_j, t_{j'}) \qquad \text{(S4)} \\ &\approx \mathrm{Cov}\left(\frac{[\mathbf{g}', \mathbf{x}'] \boldsymbol{y}_j}{\sqrt{\sigma_j^2 \sigma_g^2 (n_0 + n_1)}}, \frac{[\mathbf{g}', \mathbf{z}'] \boldsymbol{y}_{j'}}{\sqrt{\sigma_{j'}^2 \sigma_g^2 (n_0 + n_2)}} \right) \\ &= \frac{[\mathbf{g}', \mathbf{x}'] \mathrm{Cov}(\boldsymbol{y}_j, \boldsymbol{y}_{j'}) [\mathbf{g}', \mathbf{z}']'}{\sqrt{\sigma_j^2 \sigma_{j'}^2 \sigma_g^4 (n_0 + n_1)(n_0 + n_2)}} \\ &= \frac{R_{j,j'} \sigma_j \sigma_{j'} [\mathbf{g}', \mathbf{x}'] \begin{bmatrix} [0.7]\mathbf{I} & \\ & \mathbf{0} \end{bmatrix} \begin{bmatrix} [0.7]\mathbf{g} \\ \mathbf{z} \end{bmatrix}}{\sqrt{\sigma_j^2 \sigma_{j'}^2 \sigma_x^4 (n_0 + n_1)(n_0 + n_2)}} \\ &= \frac{R_{j,j'} \mathbf{g}'\mathbf{g}}{\sqrt{\sigma_g^4 (n_0 + n_1)(n_0 + n_2)}} \\ &\approx \frac{n_0}{\sqrt{(n_0 + n_1)(n_0 + n_2)}} R_{j,j'}, \qquad \text{(S5)} \end{aligned}$$

Therefore, the correlation of t-statistics is a shrinkage version of the phenotypic correlation, with a factor determined by the level of overlap. When no overlap individual exists across the phenotypes, the test statistic automatically reduces to Fisher's method of χ^2 accumulation.

In practice, it should be noted that the estimation of $\mathbf{R}^*$ using SNPs with Z-score thresholding may generate biases (Zheng et al., 2018; Shen et al., 2020). Bivariate LD score regression intercept can reduce such biases (Zheng et al., 2018) but still not the most efficient (Shen et al., 2020). Nevertheless, the small biases in the estimation of $\mathbf{R}^*$ would not alter the logic in this paper regarding different replication criteria.

Conditional multivariate analysis (cMVA)

When cMVA is performed and p SNPs $G = (G_1, ..., G_p)$ are involved, we let $\mathbf{G}_{n\times p}$ denote the genotype matrix of these p SNPs with sample size of n. Then (S1) can be extended to:

$$\boldsymbol{y}_{n\times k} = \mathbf{G}_{n\times p}\boldsymbol{\beta}'_{k\times p} + \mathbf{e}_{n\times k}. \tag{S6}$$

The effects of SNP i conditional on the other $p-1$ SNPs can be tested using

$$H_0 : \tilde{\boldsymbol{\beta}}_i = \mathbf{0}. \tag{S7}$$

where $\tilde{\boldsymbol{\beta}}_i$ represents the conditional effects of SNP i on the k traits.

Similar to (S2), above hypothesis can be tested using conditional t-test statistics from (S6). For SNP i, if the conditional t-test statistics $\tilde{\mathbf{t}}_i = [\tilde{t}_{i1}, ..., \tilde{t}_{ik}]'$ in (S6) and their correlation matrix $\tilde{\mathbf{R}}_i^*$ are available, then we can obtain the test statistic

$$\tilde{T}_i^2 = \tilde{\mathbf{t}}_i'\tilde{\mathbf{R}}_i^{*-1}\tilde{\mathbf{t}}_i, \tag{S8}$$

which also asymptotically follows a χ^2 distribution with k degrees of freedom under (S7).

Next, we will show how to get $\tilde{\mathbf{t}}_i$ and $\tilde{\mathbf{R}}_i^*$. As shown in literature (Yang et al., 2012; Ning et al., 2017), the joint regression results asymptotically only depends on: (i) the covariance structure between variants and traits; (ii) the LD structure between variants. Therefore we can approximate joint regression results including $\tilde{\mathbf{t}}_i$ using summary-level statistics from GWAS meta-analyses and a reference sample. In the following sections, we will use $\mathbf{A}_{i\cdot}$ and $\mathbf{A}_{\cdot j}$ to represent the ith row and the jth column of matrix $\mathbf{A}$ respectively, and $\mathbf{A}'_{i\cdot}$ represents $(\mathbf{A}_{i\cdot})'$. For trait j, in single trait GWAS we have

$$\begin{aligned} \hat{b}_{ij} &= (\mathbf{G}'_{\cdot i}\mathbf{G}_{\cdot i})^{-1}\mathbf{G}'_{\cdot i}\boldsymbol{y}_j \approx \frac{\mathrm{Cov}(G_i, Y_j)}{\sigma^2_{g_i}} \\ \hat{\sigma}^2_{\hat{b}_{ij}} &= \sigma^2_{r,ij}(\mathbf{G}'_{\cdot i}\mathbf{G}_{\cdot i})^{-1} \approx \frac{\sigma^2_j}{n\sigma^2_{g_i}}, \end{aligned}$$

where $\hat{b}_{ij}$ and $\hat{\sigma}^2_{\hat{b}_{ij}}$ are the estimated marginal effect of variant i on trait j and its variance, $\sigma^2_{r,ij}$ is the residual variance and can be approximated by phenotypic variance σ^2_j, and $\sigma^2_{g_i} = \mathrm{Var}(G_i)$.

The LD structure between SNPs can be approximated by a representative reference sample where individual-level genotype data are available (Yang et al., 2012). Let $\mathbf{W}$ represents the $n_W \times p$ genotypes of the reference sample, then

$$\frac{\mathbf{G}'\mathbf{G}}{n} \approx \frac{\mathbf{W}'\mathbf{W}}{n_W} \approx \mathrm{Var}(G).$$

Then the conditional effect of variant i on trait j

$$\begin{aligned}
\hat{\beta}^c_{ij} &= \left[(\mathbf{G}'\mathbf{G})^{-1}\mathbf{G}'\boldsymbol{y}_j\right]_i \\
&\approx \left[\text{Var}^{-1}(G)\text{Cov}(G, Y_j)\right]_i \\
&= \left[\text{Var}^{-1}(G)\right]_{i\cdot} \begin{bmatrix} [0.7]\sigma^2_{g_1}\hat{b}_{1j} \\ \vdots \\ \sigma^2_{g_p}\hat{b}_{pj} \end{bmatrix} \\
&= \frac{1}{\sigma_{g_i}}\left[\text{Cor}^{-1}(G)\right]_{i\cdot} \begin{bmatrix} [0.7]\sigma^{-1}_{g_1} & & \\ & \ddots & \\ & & \sigma^{-1}_{g_p} \end{bmatrix} \begin{bmatrix} [0.7]\sigma^2_{g_1}\hat{b}_{1j} \\ \vdots \\ \sigma^2_{g_p}\hat{b}_{pj} \end{bmatrix} \\
&= \frac{1}{\sigma_{g_i}}\left[\text{Cor}^{-1}(G)\right]_{i\cdot} \begin{bmatrix} [0.7]\sigma_{g_1}\hat{b}_{1j} \\ \vdots \\ \sigma_{g_p}\hat{b}_{pj} \end{bmatrix},
\end{aligned}$$

and its variance

$$\hat{\sigma}^2_{\hat{\beta}^c_{ij}} = \left[\sigma^2_{r,j}(\mathbf{G}'\mathbf{G})^{-1}\right]_{ii} \approx \frac{\sigma^2_j}{n}\left[\text{Var}^{-1}(G)\right]_{ii} = \frac{\sigma^2_j}{n\sigma^2_{g_i}}\left[\text{Cor}^{-1}(G)\right]_{ii},$$

so that the conditional t-statistic

$$\begin{aligned}
\tilde{t}_{ij} &= \frac{\hat{\beta}^c_{ij}}{\sqrt{\hat{\sigma}^2_{\hat{\beta}^c_{ij}}}} \\
&= \frac{\left[\text{Cor}^{-1}(G)\right]_{i\cdot}}{\left[\text{Cor}^{-1}(G)\right]_{ii}} \begin{bmatrix} [0.7]\frac{\sqrt{n}\sigma_{g_1}\hat{b}_{1j}}{\sigma_j} \\ \vdots \\ \frac{\sqrt{n}\sigma_{g_p}\hat{b}_{pj}}{\sigma_j} \end{bmatrix} \\
&= \frac{\left[\text{Cor}^{-1}(G)\right]_{i\cdot}}{\left[\text{Cor}^{-1}(G)\right]_{ii}} \begin{bmatrix} [0.7]\frac{\hat{b}_{1j}}{\hat{\sigma}_{\hat{b}_{1j}}} \\ \vdots \\ \frac{\hat{b}_{pj}}{\hat{\sigma}_{\hat{b}_{pj}}} \end{bmatrix} \\
&= \frac{\left[\text{Cor}^{-1}(G)\right]_{i\cdot}}{\left[\text{Cor}^{-1}(G)\right]_{ii}} \begin{bmatrix} [0.7]t_{1j} \\ \vdots \\ t_{pj} \end{bmatrix}.
\end{aligned}$$

In another word, the conditional t-statistics of SNP i are linear combinations of the marginal t-statistics of all the SNPs.

Now we focus on $\tilde{\mathbf{R}}^*_i$, which is the correlation matrix of the conditional t-statistics for SNP i. The following derivation is an extension of (S4) to (S5). Here we use $\mathbf{G}_{n_0\times p}$, $\mathbf{X}_{n_1\times p}$ and $\mathbf{Z}_{n_2\times p}$ to represent the

genotypes of shared individuals, trait j only individuals and trait j' only individuals. Then the genotypes for trait j are $[\mathbf{G}', \mathbf{X}']'$, and those for trait j' are $[\mathbf{G}', \mathbf{Z}']'$. Similar to (S3), we have

$$\frac{\mathbf{G}'\mathbf{G}}{n_0} \approx \frac{\mathbf{X}'\mathbf{X}}{n_1} \approx \frac{\mathbf{Z}'\mathbf{Z}}{n_2} \approx \text{Var}(G) \tag{S9}$$

To simplify symbols, we define $\mathbf{A}_{p\times p} = \text{Var}^{-1}(G)$, $\mathbf{B}_{p\times p} = (\mathbf{G}'\mathbf{G}+\mathbf{X}'\mathbf{X})^{-1}$ and $\mathbf{C}_{p\times p} = (\mathbf{G}'\mathbf{G}+\mathbf{Z}'\mathbf{Z})^{-1}$. Then according to (S9),

$$\mathbf{B} \approx \frac{1}{n_0+n_1}\mathbf{A}, \quad \text{and} \quad \mathbf{C} \approx \frac{1}{n_0+n_2}\mathbf{A}.$$

Therefore for a pair of traits j and j',

$$\begin{aligned}
\tilde{R}^*_{i,jj'} &= \text{Cor}(\tilde{t}_{ij}, \tilde{t}_{ij'}) = \text{Cov}(\tilde{t}_{ij}, \tilde{t}_{ij'}) & \text{(S10)}\\
&\approx \text{Cov}\left(\frac{\mathbf{B}_{i\cdot}\,[\mathbf{G}', \mathbf{X}']\,\boldsymbol{y}_j}{\sqrt{\sigma_j^2\mathbf{B}_{ii}}}, \frac{\mathbf{C}_{i\cdot}\,[\mathbf{G}', \mathbf{Z}']\,\boldsymbol{y}_{j'}}{\sqrt{\sigma_{j'}^2\mathbf{C}_{ii}}}\right)\\
&= \frac{\mathbf{B}_{i\cdot}\,[\mathbf{G}', \mathbf{X}']\,\text{Cov}(\boldsymbol{y}_j, \boldsymbol{y}_{j'})\,[\mathbf{G}', \mathbf{Z}']'\,\mathbf{C}'_{i\cdot}}{\sqrt{\sigma_j^2\sigma_{j'}^2\mathbf{B}_{ii}\mathbf{C}_{ii}}}\\
&= \frac{R_{j,j'}\sigma_j\sigma_{j'}\mathbf{B}_{i\cdot}\,[\mathbf{G}', \mathbf{X}']\begin{bmatrix}[0.7]\mathbf{I} & \\ & \mathbf{0}\end{bmatrix}\begin{bmatrix}[0.7]\mathbf{G}\\ \mathbf{Z}\end{bmatrix}\mathbf{C}'_{i\cdot}}{\sqrt{\sigma_j^2\sigma_{j'}^2\mathbf{B}_{ii}\mathbf{C}_{ii}}}\\
&= \frac{R_{j,j'}\mathbf{B}_{i\cdot}(\mathbf{G}'\mathbf{G})\mathbf{C}'_{i\cdot}}{\sqrt{\mathbf{B}_{ii}\mathbf{C}_{ii}}}\\
&\approx \frac{n_0R_{j,j'}}{\sqrt{(n_0+n_1)(n_0+n_2)}}\cdot\frac{\mathbf{A}_{i\cdot}\mathbf{A}^{-1}\mathbf{A}'_{i\cdot}}{\mathbf{A}_{ii}}\\
&= \frac{n_0}{\sqrt{(n_0+n_1)(n_0+n_2)}}R_{j,j'}. & \text{(S11)}
\end{aligned}$$

Above derivation shows that, for all SNPs, the conditional t-statistics correlation equals to the shrinkage phenotypic correlation. This means our previous $\hat{\mathbf{R}}^*$ estimated from correlating GWAS t-statistics can be used as $\tilde{\mathbf{R}}^*_i$ for any SNP in cMVA.

Correlation replication approach

For SNPs discovered in MANOVA or cMVA, to replicate their potential pleiotropic pattern in the replication sample, we develop a MC-based correlation replication approach. Following the k traits, p SNPs model in (S6), we repeatedly drew $\boldsymbol{\beta}^{MC}$ from $p \times k$ variate normal distribution $\mathcal{N}(\hat{\boldsymbol{\beta}}^c, \hat{\boldsymbol{\Sigma}})$, where $\hat{\boldsymbol{\beta}}^c = (\hat{\beta}^c_{11}, ...\hat{\beta}^c_{p1}, \hat{\beta}^c_{12}, ..., \hat{\beta}^c_{p2}, ..., \hat{\beta}^c_{1k}, ..., \hat{\beta}^c_{pk})$ is the vector of estimated conditional effects, and $\hat{\boldsymbol{\Sigma}}$ is an estimate of $\boldsymbol{\Sigma} = \text{Cov}(\hat{\boldsymbol{\beta}}^c)$. Because $\hat{\boldsymbol{\beta}}^c$ and the variances of $\{\hat{\beta}^c_{ij}\}$ can be obtained from cMVA, we only

need to estimate the elements of Cor($\hat{\boldsymbol{\beta}}^c$). Similar to (S10)-(S11),

$$\begin{aligned}
\text{Cor}(\hat{\beta}^c_{ij}, \hat{\beta}^c_{i'j'}) &= \text{Cor}(\tilde{t}_{ij}, \tilde{t}_{i'j'}) = \text{Cov}(\tilde{t}_{ij}, \tilde{t}_{i'j'}) \\
&\approx \text{Cov}\left(\frac{\mathbf{B}_{i\cdot}\,[\mathbf{G}', \mathbf{X}']\,\boldsymbol{y}_j}{\sqrt{\sigma_j^2 \mathbf{B}_{ii}}}, \frac{\mathbf{C}_{i'\cdot}\,[\mathbf{G}', \mathbf{Z}']\,\boldsymbol{y}_{j'}}{\sqrt{\sigma_{j'}^2 \mathbf{C}_{i'i'}}}\right) \\
&= \frac{\mathbf{B}_{i\cdot}\,[\mathbf{G}', \mathbf{X}']\,\text{Cov}(\boldsymbol{y}_j, \boldsymbol{y}_{j'})\,[\mathbf{G}', \mathbf{Z}']'\,\mathbf{C}'_{i'\cdot}}{\sqrt{\sigma_j^2 \sigma_{j'}^2 \mathbf{B}_{ii} \mathbf{C}_{i'i'}}} \\
&= \frac{R_{j,j'} \sigma_j \sigma_{j'} \mathbf{B}_{i\cdot}\,[\mathbf{G}', \mathbf{X}'] \begin{bmatrix} [0.7]\mathbf{I} & \\ & \mathbf{0} \end{bmatrix} \begin{bmatrix} [0.7]\mathbf{G} \\ \mathbf{Z} \end{bmatrix} \mathbf{C}'_{i'\cdot}}{\sqrt{\sigma_j^2 \sigma_{j'}^2 \mathbf{B}_{ii} \mathbf{C}_{i'i'}}} \\
&= \frac{R_{j,j'} \mathbf{B}_{i\cdot}(\mathbf{G}'\mathbf{G})\mathbf{C}'_{i'\cdot}}{\sqrt{\mathbf{B}_{ii} \mathbf{C}_{i'i'}}} \\
&= \frac{n_0 R_{j,j'}}{\sqrt{(n_0 + n_1)(n_0 + n_2)}} \cdot \frac{\mathbf{A}_{i\cdot} \mathbf{A}^{-1} \mathbf{A}'_{i'\cdot}}{\sqrt{\mathbf{A}_{ii} \mathbf{A}_{i'i'}}} \\
&\approx \frac{n_0 R_{j,j'}}{\sqrt{(n_0 + n_1)(n_0 + n_2)}} \cdot \frac{\mathbf{A}_{ii'}}{\sqrt{\mathbf{A}_{ii} \mathbf{A}_{i'i'}}},
\end{aligned}$$

which means Cor($\hat{\boldsymbol{\beta}}^c$) $\approx \hat{\mathbf{R}}^* \otimes \text{Cor}^{-1}(G)$, where $\otimes$ represents Kronecker product. Specifically, for MANOVA where $p = 1$, Cor($\hat{\boldsymbol{\beta}}^c$) $\approx \hat{\mathbf{R}}^*$.

With $\hat{\boldsymbol{\beta}}^c$ and $\hat{\boldsymbol{\Sigma}}$ for the discovery sample and the replication sample, we can draw $\boldsymbol{\beta}^{MC}_{disc}$ and $\boldsymbol{\beta}^{MC}_{rep}$, then compute their Pearson's correlation coefficient $\hat{\rho}_\beta$ and Kendall's rank correlation coefficient

$$\hat{\tau}_\beta = \frac{2}{k(k-1)} \sum_{j<j'} \text{sgn}(\beta^{MC}_{j,disc} - \beta^{MC}_{j',disc}) \cdot \text{sgn}(\beta^{MC}_{j,rep} - \beta^{MC}_{j',rep}).$$

After repeating the parametric sampling many times (10,000 times in this study), we can get an estimated distribution of ρ_β and τ_β. The parametric bootstrap confidence intervals (CI) based on this distribution can be used for inference. In this study, we reject the null hypothesis $H_0 : \tau_\beta = 0$ (or $\rho_\beta = 0$) if the lower bound of the 95% CI of parametric bootstrap distribution is larger than 0.

Constructing the new phenotype score

We construct a new phenotype score as a linear combination of the original six phenotypes, via the following multiple regression model,

$$\mathbf{g} = \mathbf{Y}\mathbf{b} + \boldsymbol{\epsilon}, \tag{S12}$$

which is equivalent to CCA.

Now we show how the coefficients estimates in (S12) can also be obtained without knowing the original data. First of all, we derive the coefficients estimates of each *swapped* GWA simple regression model,

$$\mathbf{g} = \mathbf{y}_j b^*_j + \boldsymbol{\epsilon}^*_j$$

From the summary statistics, we know the estimates of the following GWA simple regression model,

$$\mathbf{y}_j = \mathbf{g}\beta_j + \mathbf{e}_j$$

i.e.

$$\hat{\beta}_j = \frac{\mathbf{g}'\mathbf{y}_j}{\mathbf{g}'\mathbf{g}} = \frac{\mathbf{g}'\mathbf{y}_j}{2nf(1-f)}$$

assuming Hardy-Weinberg equilibrium (HWE), where f is the coding allele frequency of the marker. We also have

$$\hat{b}_j^* = \frac{\mathbf{y}_j'\mathbf{g}}{\mathbf{y}_j'\mathbf{y}_j} = \frac{\mathbf{y}_j'\mathbf{g}}{n}$$

As $\mathbf{g}'\mathbf{y}_j = \mathbf{y}_j'\mathbf{g}$, we have

$$\hat{b}_j^* = 2f(1-f)\hat{\beta}_j$$

Thereafter, when Var$(y_j) = 1$ for all j, the estimates $\hat{\mathbf{b}}$ in (S12) can be calculated as

$$\begin{aligned}\hat{\mathbf{b}} &= (\mathbf{Y}'\mathbf{Y})^{-1}\mathbf{D}\hat{\mathbf{b}}^* \\ &= \mathbf{R}^{-1}\hat{\mathbf{b}}^*\end{aligned}$$

where $\mathbf{D}$ is a diagonal matrix with the j-th element $\mathbf{y}_j'\mathbf{y}_j$. So that a new phenotype score can be defined as

$$\mathbf{S} = \mathbf{Y}\hat{\mathbf{b}}$$

The variance-covariance matrix of $\hat{\mathbf{b}}$ is

$$V(\hat{\mathbf{b}}) = \sigma_b^2(\mathbf{Y}'\mathbf{Y})^{-1}$$

where

$$\sigma_b^2 = \frac{\mathbf{g}'\mathbf{g} - \hat{\mathbf{b}}'\mathbf{D}\hat{\mathbf{b}}^*}{n-k} = \frac{2nf(1-f) - \hat{\mathbf{b}}'\mathbf{D}\hat{\mathbf{b}}^*}{n-k}$$

The standard errors of $\hat{\mathbf{b}}$ can be obtained as the square roots of the diagonal elements of $V(\hat{\mathbf{b}})$, which can be used for testing individual-phenotype associations with the SNP, corrected for all the other phenotypes as covariates.

Estimating the genetic effect on the new phenotype score

In a replication cohort, the genetic effect of each SNP on the new phenotype score S can be tested via simple regression of S on the allelic dosages g,

$$\mathbf{S} = \mathbf{g}\beta_s + \mathbf{e}_s. \tag{S13}$$

Interestingly, without knowing the original data, we can obtain the estimate of β_s in the discovery sample using summary statistics. The following proof shows that $\hat{\beta}_s$ always equals to Pillai's trace V in (S1). According to (S12), we have

$$\hat{\mathbf{b}} = (\mathbf{Y}'\mathbf{Y})^{-1}\mathbf{Y}'\mathbf{g},$$

so that in (S13),

$$\hat{\beta}_s = \frac{\mathbf{S}'\mathbf{g}}{\mathbf{g}'\mathbf{g}} = \frac{\hat{\mathbf{b}}'\mathbf{Y}'\mathbf{g}}{\mathbf{g}'\mathbf{g}} = \frac{\mathbf{g}'\mathbf{Y}(\mathbf{Y}'\mathbf{Y})^{-1}\mathbf{Y}'\mathbf{g}}{\mathbf{g}'\mathbf{g}}. \tag{S14}$$

In (S1), by definition, Pillai's trace $V = \text{tr}\{(\mathbf{T} - \mathbf{E})\,\mathbf{T}^{-1}\}$, where

$$\begin{aligned} \mathbf{E} &= \mathbf{Y}'\left\{I - \mathbf{g}\left(\mathbf{g}'\mathbf{g}\right)^{-1}\mathbf{g}'\right\}\mathbf{Y}, \\ \mathbf{T} &= \mathbf{Y}'\mathbf{Y}. \end{aligned} \tag{S15}$$

Hence

$$\mathbf{T} - \mathbf{E} = \mathbf{Y}'\mathbf{g}\left(\mathbf{g}'\mathbf{g}\right)^{-1}\mathbf{g}'\mathbf{Y}. \tag{S16}$$

Combining with (S15) and (S16), we get

$$\begin{aligned} V &= \text{tr}\{(\mathbf{T} - \mathbf{E})\,\mathbf{T}^{-1}\} \\ &= \text{tr}\left\{\frac{(\mathbf{Y}'\mathbf{g})(\mathbf{g}'\mathbf{Y})(\mathbf{Y}'\mathbf{Y})^{-1}}{\mathbf{g}'\mathbf{g}}\right\} \\ &= \text{tr}\left\{\frac{(\mathbf{g}'\mathbf{Y})(\mathbf{Y}'\mathbf{Y})^{-1}(\mathbf{Y}'\mathbf{g})}{\mathbf{g}'\mathbf{g}}\right\} \\ &= \hat{\beta}_s. \end{aligned}$$

To get the standard error of $\hat{\beta}_s$, we should notice that given the numerical equivalence of V and $\hat{\beta}_s$, $\hat{\beta}_s$ has the same distribution as V. Denote F as the F-test statistic in (S1), V can be transformed as

$$F = \frac{V^2/k}{(1 - V^2)/(n - k - 1)}.$$

After rearranging,

$$\hat{\beta}_s = V = \frac{kF}{(n - k - 1) + kF}.$$

As $F \sim F(k, n - k - 1)$, we have

$$\hat{\beta}_s = V \sim Beta\left(\frac{k}{2}, \frac{n - k - 1}{2}\right),$$

which is the exact distribution of $\hat{\beta}_s$. In practice, the standard error of $\hat{\beta}_s$ can be obtained by Gaussian approximation of the Beta distribution. If we translate the MANOVA p-value back to a 1 d.f. χ^2 statistic C, we have

$$\frac{\hat{\beta}_s^2}{\text{Var}(\hat{\beta}_s)} = \frac{V^2}{\text{Var}(\hat{\beta}_s)} \approx C.$$

Then we can compute the standard error of $\hat{\beta}_s$ in the meta-GWAS population as $VC^{-1/2}$.

Also, if we denote R^2 as the coefficient of determination of both regressions (S12) and (S13) in the discovery sample, we have

$$\begin{aligned}
R^2 &= \frac{\hat{\beta}_s' \mathbf{g}'\mathbf{g}\hat{\beta}_s}{\mathrm{s}'\mathrm{s}} = \frac{\hat{\beta}_s^2 \mathbf{g}'\mathbf{g}}{\hat{\mathbf{b}}'\mathbf{Y}'\mathbf{Y}\hat{\mathbf{b}}} \\
&= \frac{\hat{\beta}_s^2 \mathbf{g}'\mathbf{g}}{\mathbf{g}'\mathbf{Y}(\mathbf{Y}'\mathbf{Y})^{-1}\mathbf{Y}'\mathbf{Y}(\mathbf{Y}'\mathbf{Y})^{-1}\mathbf{Y}'\mathbf{g}} \\
&= \frac{\hat{\beta}_s^2 \mathbf{g}'\mathbf{g}}{\mathbf{g}'\mathbf{Y}(\mathbf{Y}'\mathbf{Y})^{-1}\mathbf{Y}'\mathbf{g}} \\
&= \frac{\hat{\beta}_s^2}{\left\{\mathbf{g}'\mathbf{Y}(\mathbf{Y}'\mathbf{Y})^{-1}\mathbf{Y}'\mathbf{g}\right\}/(\mathbf{g}'\mathbf{g})} \\
&= \frac{\hat{\beta}_s^2}{\hat{\beta}_s} \\
&= \hat{\beta}_s
\end{aligned}$$

Therefore, Pillai's trace V directly represents the proportion of the variance of S explained by the SNP.

1.2 Simulation settings

100 simulations were performed for each scenario. In each simulation, a discovery sample and a replication sample were generated with one SNP and either 6 or 32 traits. For simplicity, the genotypes g were simulated from a standard normal distribution. This provides the same sufficient statistics as if g came from a binomial distribution $B(2, f)$ assuming Hardy-Weinberg equilibrium, where f is the minor allele frequency, while standardizing the mean from $2f$ to zero and variance from $2f(1-f)$ to one. In the following sections, we denote the cross-phenotype effects of the SNP on the k traits as $\boldsymbol{\beta} = (\beta_1, ..., \beta_k)$, and the residuals as $\boldsymbol{\epsilon} = (\epsilon_1, ..., \epsilon_k)$. As we demonstrate the replication strategies for *single*-SNP multi-trait tests, linkage disequilibrium (LD) between SNPs are not considered in the simulation. In practice, the $\boldsymbol{\beta}$ values are usually not the true causal effects but rather the tagged effects by the SNP due to its LD with the causal variant(s).

Phenotypes $\mathbf{y} = \boldsymbol{\beta} g + \boldsymbol{\epsilon}$. To differentiate the cross-phenotype effects in discovery sample from those in replication sample, we use $\boldsymbol{\beta}_{disc}$ and $\boldsymbol{\beta}_{rep}$ to represent them respectively. Similarly, for residuals, we use $\boldsymbol{\epsilon}_{disc}$ and $\boldsymbol{\epsilon}_{rep}$.

6 traits settings

For both the discovery and replication cohorts, genotypes and phenotypes for 2,000 independent individuals were simulated. The real phenotypic correlation matrices Σ_{GIANT} and Σ_{UKB} between six anthropometric traits (Body mass index, Height, Weight, Hip circumference, Waist-hip ratio and Waist circumference) in GIANT and UKB respectively were involved in this simulation.

$\boldsymbol{\beta}_{disc}$ and $\boldsymbol{\beta}_{rep}$ under different scenarios were simulated as below. (i) Null: $\boldsymbol{\beta}_{disc} = \boldsymbol{\beta}_{rep} = \mathbf{0}$. (ii) Unmatched multi-trait effects: $\beta_{i,disc} \sim N(0, 10^{-4})$, $i = 1, ..., 6$, and $\beta_{i,rep}$, $i = 1, ..., 6$ were independently drawn from $N(0, 10^{-4})$. (iii) Matched single-trait effect: $\beta_{1,disc} \sim N(0, 2 \times 10^{-4})$ and $\beta_{1,rep} = \beta_{1,disc}$, while $(\beta_{2,disc}, ..., \beta_{6,disc}) = (\beta_{2,rep}, ..., \beta_{6,rep}) = \mathbf{0}$. (iv) Matched multi-trait effects: $\beta_{i,disc} \sim N(0, 10^{-4})$, $i = 1, ..., 6$, and $\boldsymbol{\beta}_{rep} = \boldsymbol{\beta}_{disc}$.

For "consistent R" scenario, both $\boldsymbol{\epsilon}_{disc}$ and $\boldsymbol{\epsilon}_{rep}$ follow $\mathcal{N}(\mathbf{0}, \Sigma_{\text{GIANT}})$. For "different R" scenario, $\boldsymbol{\epsilon}_{disc} \sim \mathcal{N}(\mathbf{0}, \Sigma_{\text{GIANT}})$ and $\boldsymbol{\epsilon}_{rep} \sim \mathcal{N}(\mathbf{0}, \Sigma_{\text{UKB}})$.

32 traits settings

For both the discovery and replication cohorts, genotypes and phenotypes for 4,000 independent individuals were simulated. The real phenotypic correlation matrix $\Sigma_{\text{UKB, 32traits}}$ between 32 anthropometric traits in UKB was involved in this simulation. The 32 traits are: Weight, Body mass index (BMI), Leg fat-free mass (right), Leg predicted mass (left), Leg predicted mass (right), Arm fat percentage (left), Arm fat percentage (right), Arm fat mass (left), Arm fat mass (right), Arm fat-free mass (right), Arm fat-free mass (left), Arm predicted mass (left), Arm predicted mass (right), Trunk fat percentage, Trunk fat mass, Trunk fat-free mass, Trunk predicted mass, Basal metabolic rate, Body fat percentage, Whole body fat mass, Whole body fat-free mass, Whole body water mass, Leg fat percentage (left), Leg fat percentage (right), Leg fat mass (left), Leg fat mass (right), Leg fat-free mass (left), Impedance of whole body, Impedance of arm (left), Impedance of arm (right), Impedance of leg (left) and Impedance of leg (right).

$\boldsymbol{\beta}_{disc}$ and $\boldsymbol{\beta}_{rep}$ under different scenarios were simulated as below. (i) Null: $\boldsymbol{\beta}_{disc} = \boldsymbol{\beta}_{rep} = \mathbf{0}$. (ii) Unmatched multi-trait effects: $\beta_{i,disc} \sim N(0, 10^{-4})$, $i = 1, ..., 32$, and $\beta_{i,rep}$, $i = 1, ..., 32$ were independently drawn from $N(0, 10^{-4})$. (iii) Matched single-trait effect: $\beta_{1,disc} \sim N(0, \times 10^{-2})$ and $\beta_{1,rep} = \beta_{1,disc}$, while $(\beta_{2,disc}, ..., \beta_{32,disc}) = (\beta_{2,rep}, ..., \beta_{32,rep}) = \mathbf{0}$. (iv) Matched multi-trait effects: $\beta_{i,disc} \sim N(0, 10^{-4})$, $i = 1, ..., 32$, and $\boldsymbol{\beta}_{rep} = \boldsymbol{\beta}_{disc}$.

Because the phenotypic correlations between some traits among the 32 are very close to 1, we generated two shrinkage correlation matrices based on the original $\Sigma_{\text{UKB, 32traits}}$. The off-diagonal elements in the first matrix $\boldsymbol{\Sigma}_1$ are half of their correspondences in $\Sigma_{\text{UKB, 32traits}}$. Then R function "nearPD" was used to guarantee the matrix is positive-definite. In the second matrix $\boldsymbol{\Sigma}_2$, the 32 traits were split into two groups. For group 1, the off-diagonal elements equal to their correspondences in $\boldsymbol{\Sigma}_1$; for group 2, the off-diagonal elements equal to their correspondences in $\boldsymbol{\Sigma}_1$ plus random terms drawn from $U(-0.1, 0.1)$; and for the correlations between group 1 and 2, the elements equal to their correspondences in $\boldsymbol{\Sigma}_1$ divided by $\sqrt{3}$. Then R function "nearPD" was used to guarantee the matrix is positive-definite. For "consistent R" scenario, both $\boldsymbol{\epsilon}_{disc}$ and $\boldsymbol{\epsilon}_{rep}$ follow $\mathcal{N}(\mathbf{0}, \boldsymbol{\Sigma}_1)$. For "different R" scenario, $\boldsymbol{\epsilon}_{disc} \sim \mathcal{N}(\mathbf{0}, \boldsymbol{\Sigma}_1)$ and $\boldsymbol{\epsilon}_{rep} \sim \mathcal{N}(\mathbf{0}, \boldsymbol{\Sigma}_2)$.

2 SUPPLEMENTARY TABLES AND FIGURES

2.1 Tables

Table S1. Summary of 24 loci detected and replicated by MANOVA for six anthropometric traits.

Top variant	Nearest genes	EA	p_G	p_U	$\beta_{S,G}$	$\beta_{S,U}$	$p_{S,U}$	ρ_β	CI (ρ_β)	τ_β	CI (τ_β)
rs2138275	FANK1	T	2.41E-08	1.96E-05	2.13E-04	2.24E-04	1.97E-08	*1	[0.88, 1]	*1	[0.73, 1]
rs4646404	PEMT	A	1.08E-10	1.54E-07	3.93E-04	3.26E-04	7.30E-10	*0.96	[0.72, 1]	*1	[0.6, 1]
rs1458758	NUDT6	T	3.45E-13	9.39E-11	3.46E-04	4.03E-04	6.10E-14	*0.99	[0.87, 1]	*0.96	[0.6, 1]
rs12744534	PRRX1	A	5.11E-11	5.10E-09	3.83E-04	3.36E-04	1.43E-11	*0.93	[0.7, 1]	*0.73	[0.6, 1]
rs9991328	FAM13A	T	1.38E-09	7.46E-15	3.11E-04	4.08E-04	1.23E-16	*0.98	[0.84, 1]	*0.86	[0.46, 1]
rs972283	LOC105375508	A	1.74E-10	5.75E-12	2.99E-04	2.31E-04	2.22E-11	*0.86	[0.53, 1]	*0.86	[0.46, 1]
rs10761785	REEP3	T	1.19E-10	5.47E-10	3.02E-04	3.58E-04	7.40E-13	*0.98	[0.88, 1]	*0.86	[0.46, 1]
rs12454712	BCL2	T	2.64E-09	9.51E-09	3.11E-04	3.06E-04	8.36E-12	*0.93	[0.58, 1]	*0.86	[0.46, 1]
rs905938	ZBTB7B	T	8.09E-16	3.39E-11	5.33E-04	5.43E-04	7.73E-15	*0.99	[0.87, 1]	*0.82	[0.46, 1]
rs2925979	CMIP	T	5.88E-11	1.42E-11	2.96E-04	2.99E-04	2.72E-12	*0.90	[0.65, 1]	*0.82	[0.46, 1]
rs6090583	EYA2	A	2.27E-11	1.07E-10	3.98E-04	3.58E-04	1.35E-10	*0.90	[0.74, 1]	*0.60	[0.46, 1]
rs11974409	TBL2	A	6.94E-09	4.93E-06	2.19E-04	2.18E-04	4.17E-09	*0.96	[0.71, 1]	*0.86	[0.33, 1]
rs17819328	TSEN2	T	8.58E-15	8.20E-12	4.96E-04	4.25E-04	6.69E-14	*0.87	[0.66, 1]	*0.60	[0.33, 1]
rs1053593	HMGXB4	T	1.26E-09	4.62E-05	3.19E-04	2.00E-04	3.66E-05	*0.94	[0.5, 1]	*0.60	[0.33, 1]
rs6780459	LOC107986108	A	8.30E-09	7.39E-06	2.51E-04	1.74E-04	1.06E-06	*0.87	[0.61, 1]	*0.46	[0.33, 1]
rs486359	SLC22A3	C	2.05E-12	2.55E-11	3.26E-04	3.70E-04	1.27E-12	*0.86	[0.58, 1]	*0.73	[0.2, 1]
rs1045241	TNFAIP8	T	1.24E-08	5.12E-06	2.96E-04	2.11E-04	3.80E-06	*0.88	[0.61, 1]	*0.60	[0.2, 1]
rs12608504	IQCN	A	5.15E-09	1.10E-07	2.89E-04	2.09E-04	7.96E-07	*0.70	[0.3, 0.99]	*0.60	[0.2, 1]
rs11231693	FLRT1	A	1.21E-09	1.97E-05	2.35E-04	1.04E-04	5.03E-03	*0.59	[0.16, 1]	*0.33	[0.2, 1]
rs2278557	PPP4C	C	9.15E-10	2.21E-13	3.72E-04	4.58E-04	3.87E-15	*0.68	[0.01, 1]	*0.69	[0.06, 1]
rs823114	NUCKS1	A	3.80E-08	9.99E-15	1.98E-04	1.81E-04	5.37E-07	0.61	[-0.25, 0.99]	0.60	[-0.07, 1]
rs459552	APC	A	1.44E-08	1.45E-05	2.63E-04	2.24E-04	5.82E-07	0.73	[-0.19, 1]	0.60	[-0.07, 1]
rs4968164	VPS53	A	2.38E-08	5.00E-09	4.09E-04	2.09E-04	3.39E-05	0.44	[-0.07, 0.99]	0.46	[-0.07, 1]
rs9294260	LOC105377876	A	2.31E-08	2.47E-05	2.13E-04	5.24E-05	5.80E-03	0.18	[-0.49, 0.95]	-0.20	[-0.47, 0.87]

EA, effect allele; p_G, MANOVA p-value using summary statistics from GIANT; p_U, MANOVA p-value using individual-level data from UKB; $\beta_{S,G}$, the estimated effect of the SNP on phenotype-score in GIANT; $\beta_{S,U}$, the estimated effect of the SNP on phenotype-score in UKB; $p_{S,U}$, the p-value of phenotype-score replication in UKB; ρ_β, the observed Pearson's correlation coefficient of multivariate marginal effects between GIANT and UKB, significant results are asterisked ; CI (ρ_β), the 95% confidence interval of the empirical distribution for ρ_β; τ_β, the observed Kendall's rank correlation coefficient of multivariate marginal effects between GIANT and UKB, significant results are asterisked ; CI (τ_β), the 95% confidence interval of the empirical distribution for τ_β.

Table S2. Correlation matrix of the involved anthropometric traits in GIANT and UKB.

2.2 Figures

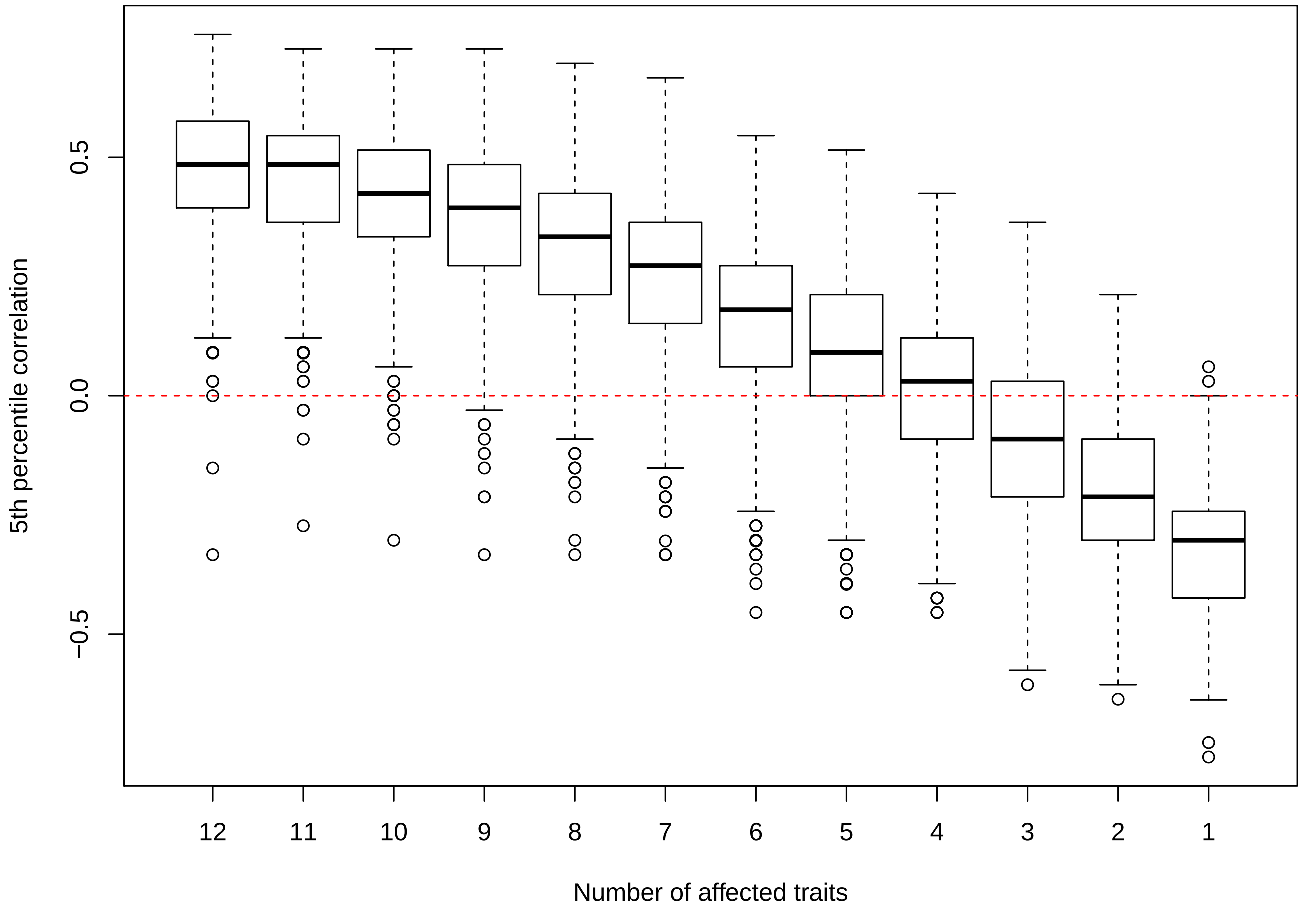

Figure S1. The performance of correlation replication when zero effect sizes exist. In this simulation, we set the marginal effects of a SNP on 12 traits in discovery and replication sample to be same. We firstly simulated 1,000 groups of marginal effects. In each group, 12 coefficients were drawn from $\mathcal{N}(0,1)$, which are the marginal effects of a SNP on 12 traits in discovery and replication sample. Because the effect sizes for each trait are same across two samples, the true $\tau_\beta = 1$. To simulate the impact of zero effect sizes on the MC-based distribution of τ_β in correlation test, we set the first several effect sizes as zero. In this case, the true $\tau_\beta = 1$ still, but the MC-based distribution of τ_β would change. We then simulated a SNP for 10,000 individuals. The SNP explains 0.1% variance of each trait. The phenotypic correlation matrix of the 12 simulated traits is set as a block diagonal matrix, where the first 6×6 is the estimated shrinkage phenotypic correlation matrix from GIANT and the second 6×6 is the phenotypic correlation matrix from UKB. After this, we sampled one group of coefficients from the 1,000 groups and simulated phenotypes. Then we performed the replication test and got the parametric bootstrap distribution of τ_β. The x-axis represents the number of traits on which the SNP has non-zero effect. The y-axis is the 5th percentile of the MC-based distribution of τ_β.

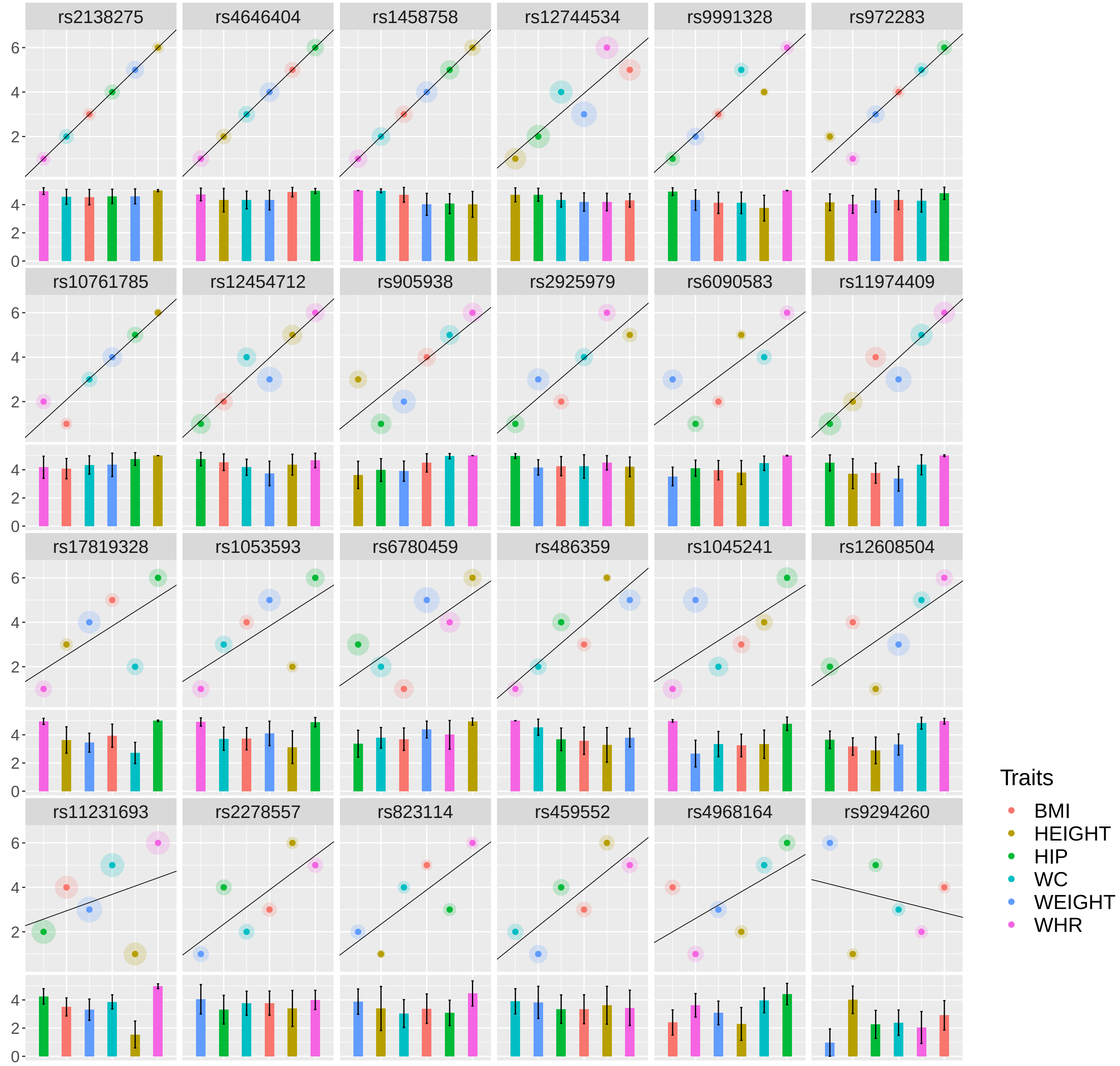

Figure S2. The Kendall's correlations of the estimated marginal effects from GIANT and UKB at 24 loci which are replicated by six-traits MVA. The panels are reordered in descending order according to the lower bounds of their 95% CI in correlation replication. Each color represents one trait. There are two parts in each panel. In both parts, the x-axis is the ranks of estimated marginal effect sizes in ascending order from GIANT. For the upper part, the y-axis is the ranks from UKB. Therefore each dot represents the rank in GIANT and UKB for one trait. The radius of shade around a dot is proportional to the standard error of the estimated marginal effect. The standard errors are computed with variances in GIANT and UKB using inverse variance weights. To facilitate visualization, a regression line is added. Its slope equals to the Spearman's correlation. The lower part shows the results based on 10,000 times Monte-Carlo simulations (described in the Materials and Methods). The y-axis is the mean number of concordant pairs generated by a trait. If a trait has a very low bar, it means the trait disturbs the consistency. The whiskers represent $\pm$ 1 times the standard deviation about the mean.

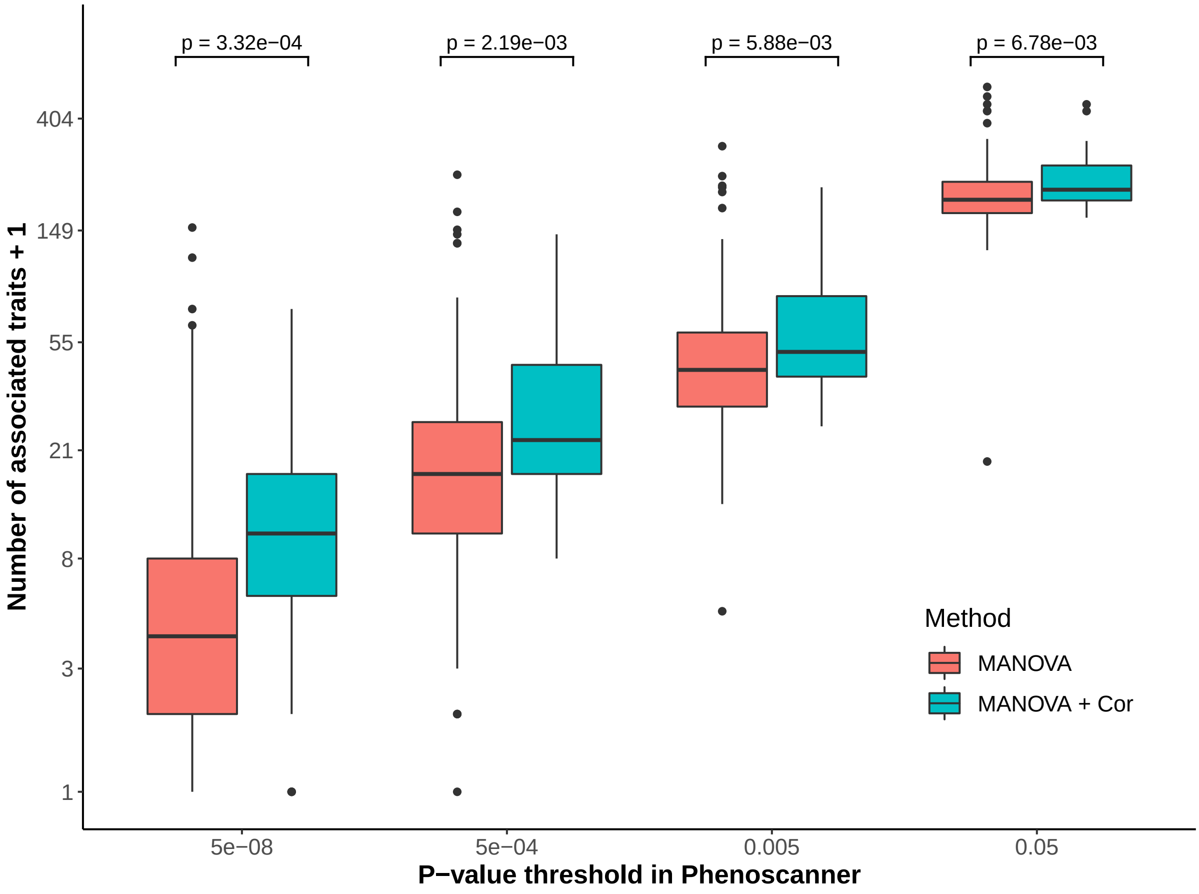

Figure S3. The number of associated traits across different PhenoScanner p-value threshold. The x-axis represents the p-value threshold in PhenoScanner; the y-axis is the number of associated traits plus one in logarithmic scale. MANOVA (Red boxes): all the 317 loci replicated in UKB by MANOVA with top SNP MANOVA p-value $< 0.05/449$; MANOVA + Cor (blue boxes): 32 loci (among the above 317 loci) with the lower bounds of 95% CI higher or equal to 0.73 in correlation replication. The p-values in the figure were computed from the Wilcoxon signed-rank test.

REFERENCES

Ning, Z., Lee, Y., Joshi, P. K., Wilson, J. F., Pawitan, Y., and Shen, X. (2017). A selection operator for summary association statistics reveals allelic heterogeneity of complex traits. *The American Journal of Human Genetics* 101, 903–912

Olson, C. L. (1976). On choosing a test statistic in multivariate analysis of variance. *Psychological Bulletin* 83, 579

Shen, X., Li, T., and Ning, Z. (2020). Improved estimation of phenotypic correlations using summary association statistics. *bioRxiv* 0, 0–0. doi:https://doi.org/10.1101/2020.12.10.419325

Yang, J., Ferreira, T., Morris, A. P., Medland, S. E., Genetic Investigation of ANthropometric Traits (GIANT) Consortium, DIAbetes Genetics Replication And Meta-analysis (DIAGRAM) Consortium, et al. (2012). Conditional and joint multiple-SNP analysis of GWAS summary statistics identifies additional variants influencing complex traits. *Nature genetics* 44, 369–75– S1–3

Zheng, J., Richardson, T. G., Millard, L. A. C., Hemani, G., Elsworth, B. L., Raistrick, C. A., et al. (2018). PhenoSpD: an integrated toolkit for phenotypic correlation estimation and multiple testing correction

using GWAS summary statistics. *GigaScience* 7, 1–10. doi:10.1093/gigascience/giy090

Zhu, X., Feng, T., Tayo, B. O., Liang, J., Young, J. H., Franceschini, N., et al. (2015). Meta-analysis of correlated traits via summary statistics from GWASs with an application in hypertension. *American journal of human genetics* 96, 21–36
